# Patient experiences and unmet needs in diagnosis and treatment of metastatic colorectal cancer: results of a US survey

**DOI:** 10.1080/14796694.2026.2695213

**Published:** 2026-10-10

**Authors:** Kim Newcomer, Julie Clauer, Khadijah Ameen, Andrea Incudine, Amy Mulvey, Kathy Steinberg, Jeneth Aquino, Ashley Geiger, Scott Campbell, Ashley Calabrese, Mohamedtaki Tejani, Manish Shah

**Affiliations:** a Colorectal Cancer Alliance, Washington, DC, USA; b COLONTOWN, Crownsville, MD, USA; c BLKHLTH, Atlanta, GA, USA; d Family Reach, Boston, MA, USA; e The Harris Poll, Chicago, IL, USA; f Vanderbilt University Medical Center, Nashville, TN, USA; g Takeda Development Center Americas, Inc. (TDCA), Cambridge, MA, USA; h Takeda Pharmaceuticals U.S.A., Inc., Cambridge, MA, USA; i Advent Health Cancer Institute, Orlando, FL, USA; j Weill Cornell Medicine and New York Presbyterian, New York, NY, USA

**Keywords:** Financial burden, metastatic colorectal cancer, patient experience, quality of life, shared decision-making, survey, treatment, United States

## Abstract

**Background:**

Metastatic colorectal cancer (mCRC) can impact patients beyond their physical health. We conducted a survey to evaluate patient perspectives on care and treatment experience.

**Methods:**

The survey was conducted by The Harris Poll in the United States. Adults with mCRC were recruited via online panels and patient advocacy groups. Sampling precision was measured via a Bayesian credible interval; data were accurate to within 5.3% points (95% confidence level).

**Results:**

Of the 344 respondents, 46% identified as non-Hispanic White, 25% as non-Hispanic Black/African American, and 22% as Hispanic; the mean age was 56 years. Overall, 31% reported that their mCRC diagnosis was delayed, with 83% wishing more information had been provided by their health-care provider at diagnosis. Mental health resources were difficult to access for 53% of the respondents and 88% experienced financial impacts because of their mCRC diagnosis. Furthermore, 49% reported negative impacts from health inequities affecting their care. Overall, 67% of the respondents wished for more power in treatment decision-making.

**Conclusions:**

The survey highlights key unmet medical and non-medical patient needs in mCRC care. Access to informational resources, including for financial assistance, and a greater emphasis on shared treatment decision-making would help patients feel valued and improve their mCRC experience.

## PLAIN LANGUAGE SUMMARY

What is this paper about?

Cancer of the colon or rectum that has spread to other organs in the body, known as metastatic colorectal cancer (mCRC), can affect people in ways beyond their physical health. We created a 60-question online survey to better understand the experiences of adults living with mCRC across the United States (US).

What were the results?

The survey was completed by 344 people with mCRC. Nearly 1 in 3 people who responded (31%) felt there was a delay in their diagnosis with mCRC. Most patients (83%) also wished they had more information shared with them about living with mCRC when they were diagnosed.

Among other major challenges identified, many patients said it was difficult to find the mental health resources they needed. Patients also felt that their diagnosis had a negative effect on their financial wellbeing, and some felt that they had been affected by unequal access to care due to discrimination. Two-thirds wished they had more power in decisions about their treatment.

What do the results mean?

These results highlight important challenges for people living with mCRC in the US, which can affect many aspects of their daily lives. Life with mCRC could be improved if people had easier access to supportive services and financial help, and if more focus was placed on patients and health-care teams making treatment decisions together. Understanding these common challenges may help patients feel less alone and support more open conversations with their health-care teams about what they need.

## Graphical abstract

**Figure:** Video abstract The written transcript can be accessed in the supplementary material https://www.tandfonline.com/doi/suppl/10.1080/14796694.2026.2695213.

## 1. Introduction

Globally, colorectal cancer (CRC) is the third most diagnosed cancer [1]; in the United States (US), almost 1.4 million people are currently living with CRC [2], with more than 154,000 new cases and almost 53,000 deaths projected in 2025 alone [3]. Approximately 20% of patients present with metastatic disease at initial CRC diagnosis globally [4], while almost 50% of patients go on to develop metastases during the course of their disease [5].

CRC incidence and survival vary substantially depending on patient characteristics [6]. For example, Black patients are more likely to experience delayed diagnosis, with 25% of Black patients having metastatic CRC (mCRC) at the initial diagnosis compared with 21% of White patients [6]. Additionally, Black/African American populations are around 20% more likely to develop CRC, and have around 40% increased mortality compared with White populations [7]. The overall 5-year CRC survival rates for Black patients have consistently been 6–12% lower than for White patients, with a notable difference in mCRC rates (12% versus 17%) [8]. Additionally, the incidence of CRC in Hispanic young adults (aged <50 years) has also seen a greater increase compared with White and African American young adults: between 1993 and 2007, incidence rates of CRC increased by 45% in Hispanic young adults, compared with 27% in White and 15% in African American young adults [9]. The factors that contribute to these health-care disparities among patients with CRC are complex. Socioeconomic considerations, such as income, education level, and occupational status impact access to health-care services and resources, and may disproportionately affect certain racial/ethnic groups [10].

Over the past 30 years, the median overall survival for patients with mCRC has steadily increased from 14 months [11] to 32 months, with many patients now living for 5 years with metastatic disease [12]. This improvement in survival is largely attributable to advances in therapy and multidisciplinary care. Despite the increased availability of treatment options for mCRC [13], overcoming resistance and managing treatment-related effects remain an issue.

Patients with CRC experience a high symptom burden that can persist after treatment [14]. These symptom experiences include fatigue, nausea, diarrhea, constipation, and pain [15], which impair patients’ physical, social, and psychological quality of life (QoL) [16,17].

With an extended overall survival and increased symptoms from both the disease and its treatment, patients with mCRC experience a greater burden of their illness, which can also affect the experience of caregivers. To improve patients’ QoL and optimize outcomes, it is critical to gain insights into the experiences and challenges faced by patients. We performed an online patient survey conducted by The Harris Poll in collaboration with leading patient advocacy groups (PAGs) who support patients with cancer to better understand the patient perspective of living with mCRC. The survey explored the patient experience relating to health equity, QoL, treatment and decision making, and financial health, with the aim of identifying opportunities to improve patient care, through awareness, education, information provision, and financial assistance.

## 2. Methods

### 2.1. Survey design

The survey was conducted in collaboration with BLKHLTH, COLONTOWN, Colorectal Cancer Alliance, and Family Reach. Involvement of these four PAGs in survey development ensured that the questions and response options were worded appropriately and were reflective of the patient experience. The survey comprised 60 questions in English, which explored patient profile (20 items), diagnosis experience (8 items), the role of PAGs/other resources (4 items), QoL (2 items), access to care and financial challenges (12 items), health equity (4 items), and treatment experience (10 items). Most questions were asked as either binary (yes/no answers) or on a Likert scale (e.g., relating to level of agreement [strongly agree, somewhat agree, somewhat disagree, and strongly disagree] or the degree to which something described the patient experience [very well, somewhat well, not very well, and not at all well]). This self-reported survey was conducted online, with an estimated completion time of 15 min. Typically, survey research submitted to the Institutional Review Board (IRB) for review is recognized as exempt; therefore, a formal IRB review was not sought for this survey [18]. The survey readership was taken into account when drafting the questions, all data were collected anonymously, only aggregate data are reported, and informed consent was sought.

### 2.2. Participants

Eligible patients were self-identified adults aged ≥18 years who had been diagnosed with stage 4 mCRC and were residing in the US at the time of the survey. Patients were recruited through online panels and PAGs. Prospective participants were sent an invitation with a link to the online survey and were required to provide consent and complete a survey screener to confirm that they met the qualification criteria before being allowed to proceed. Participants recruited via online panels were offered a nominal honorarium in the form of redeemable incentivized points. Participants recruited via PAGs were offered a $25 incentive, either via a gift card or charity donation. A variety of in-survey checks were performed during fielding to ensure the quality of the final results (e.g., monitoring for illogical response combinations).

### 2.3. Data collection

The online survey was conducted between 17 January and 7 February 2024. Demographic information collected for each respondent included gender, current age, race/ethnicity, residence by region in the US, income, marital status, employment status, parental status, education level, and household size. The survey explored patients’ diagnostic journeys and treatment experiences, with the data collected representing patient perceptions, as well as factors that patients felt could have changed their healthcare experience.

Invitations to the online survey were sent to prospective respondents by partner organizations. Prospective respondents who were recruited through PAGs were asked to provide consent and to complete the survey screener to confirm that they met the required qualification criteria and to collect the contact details required to distribute incentives; contact details (including names and e-mail addresses) were collected by The Harris Poll and connected with respondent data for incentive distribution, although this information was never shared with the survey sponsor. For respondents recruited via panel partners, personally identifiable information (PII) collected via the survey screener was stored in a secure database, in accordance with all local laws and legal regulations. This PII database was not connected to any survey data; instead, unique identifiers were used to identify respondents who had completed the survey, which allowed the survey partners to distribute incentives. Survey data were aggregated for reporting and analysis.

### 2.4. Data analysis

The sampling precision of Harris online polls is measured using a Bayesian credible interval. For this survey, Quantum software was used for statistical analysis, and data were accurate to within 5.3% points using a student’s t-test at a 95% confidence level. Results were analyzed for the overall survey population, as well as for patient subgroups, including race/ethnicity (non-Hispanic Black/African American, Hispanic, non-Hispanic White), age (18–44 years, 45–64 years, ≥65 years), and gender (male, female). Where the base size fell below *n* = 100, results should be interpreted directionally only. The credible interval was wider among subgroups of the survey population. Raw data were not weighted and therefore represent survey respondents only.

## 3. Results

### 3.1. Participation rates

Of the 9561 patients invited, 1240 completed the survey for a participation rate of 13%. Of these, 896 patients did not meet qualification criteria and were excluded, including 173 who only partially completed the survey (Supplementary Figure S1).

### 3.2. Patient demographics and characteristics

Of the 344 respondents who met the qualification criteria and completed the survey, 254 were recruited via online panels and 90 via PAGs. The mean age was 56 years (standard deviation [SD]: 13) and 55% (*n* = 190) were male (Table 1). In terms of race/ethnicity, 46% of the respondents identified as non-Hispanic White (*n* = 158), 25% as non-Hispanic Black/African American (*n* = 87), 22% as Hispanic (*n* = 75), 5% as Asian (*n* = 17), 1% as Native Hawaiian or other Pacific Islander (*n* = 3) and 1% as American Indian or as Alaska Native (*n* = 2). Among Hispanic respondents, 16% (*n* = 12) also identified as White and 72% (*n* = 54) as Black/African American. Overall physical health was reported as being poor by 67% (*n* = 232) of respondents, fair by 17% (*n* = 59), good by 13% (*n* = 44), and excellent by 3% (*n* = 9). Among those who had reported receiving treatment (74%; *n* = 256), the most common types reported were chemotherapy (66%, *n* = 169), surgery (57%, *n* = 147), and radiation therapy (39%, *n* = 101). Most commonly, respondents had an annual household income of between $100,000–$149,999 (43%; *n* = 148) (Supplementary Table 1). The highest level of education was a 4-year college degree or above for 47% of the respondents (*n* = 161), with 46% (*n* = 158) obtaining a high-school diploma and 7% (*n* = 25) less than a high-school diploma.

**Table 1.** Patient demographics and perceptions of disease characteristics.

Characteristic, *n* (%)	All respondents (*N* = 344)	Non-Hispanic Black/African American respondents (*n* = 87)*	Non-Hispanic White respondents (*n* = 158)	Hispanic respondents (*n* = 75)*^†^
**Gender**				
Male	190 (55)	54 (62)	71 (45)	54 (72)
Female	154 (45)	33 (38)	87 (55)	21 (28)
**Age, years**				
18–44	77 (22)	18 (21)	32 (20)	24 (32)
45–64	161 (47)	47 (54)	81 (51)	28 (37)
65+	106 (31)	22 (25)	45 (28)	23 (31)
**Current overall physical health**				
Good/excellent	53 (15)	1 (1)	46 (29)	2 (3)
Poor/fair	291 (85)	86 (99)	112 (71)	73 (97)
**Cancer diagnosis**				
Colorectal	286 (83)	73 (84)	116 (73)	73 (97)
Colon	60 (17)	9 (10)	44 (28)	4 (5)
Rectal	25 (7)	6 (7)	17 (11)	0
**Stage at diagnosis**				
1	56 (16)	20 (23)	20 (13)	10 (13)
2	70 (20)	15 (17)	36 (23)	15 (20)
3	120 (35)	31 (36)	39 (25)	43 (57)
4	88 (26)	16 (18)	59 (37)	7 (9)
**Types of mCRC treatment respondents reported receiving**^‡^				
Chemotherapy	169 (66)	23 (44)	104 (81)	33 (55)
Surgery	147 (57)	23 (44)	86 (67)	32 (53)
Radiation therapy	101 (39)	25 (48)	52 (40)	15 (25)
Immunotherapy	72 (28)	16 (31)	30 (23)	20 (33)
Targeted therapy	61 (24)	12 (23)	30 (23)	14 (23)
Other	13 (5)	0	12 (9)	0

*Base sizes are <100; therefore, results should only be interpreted directionally. ^†^Among Hispanic respondents, 16% (*n* = 12) also identified as White and 72% (*n* = 54) as Black/African American. ^‡^Among patients who reported ever having received mCRC treatment.

mCRC, metastatic colorectal cancer.

### 3.3. Diagnosis experience

At the time of the initial diagnosis, 16% (*n* = 56) of respondents reported having stage 1 CRC. Most commonly, respondents (35%, *n* = 120) reported having stage 3 CRC at the time of first diagnosis, including 57% (*n* = 43) of Hispanic, 25% (*n* = 39) of non-Hispanic White, and 36% (*n* = 31) of non-Hispanic Black respondents. Respondents’ mean age was 50 (SD: 12) years at symptom onset, 52 (SD: 12) years at first diagnosis, and 56 (SD: 12) years for those who were restaged to stage 4 mCRC. The mean reported time from symptom onset to initial diagnosis was 1.4 (SD: 1.7) years for all respondents; 1.7 years for non-Hispanic Black respondents; 1.4 years for non-Hispanic White respondents; and 0.7 years for Hispanic respondents. Among all respondents, 31% (*n* = 105) felt their diagnosis was delayed; this included 21% (*n* = 18) of non-Hispanic Black, 34% (*n* = 53) of non-Hispanic White, and 41% (*n* = 31) of Hispanic respondents. In addition, a higher proportion of females (38%, *n* = 59) than males (24%, *n* = 46), and of respondents aged ≤44 years (47%, *n* = 36) than >44 years (26%, *n* = 69) also believed that they had experienced a delay in diagnosis. Factors that respondents felt could have contributed to a quicker diagnosis are shown in Figure 1 and included having a greater awareness of mCRC, trustworthy informational resources, and easy-to-understand informational resources. Overall, 62% (*n* = 54) of non-Hispanic Black, 43% (*n* = 32) of Hispanic, and 38% (*n* = 60) of non-Hispanic White respondents believed that a greater availability of appropriate informational resources that were easier to understand and that they felt they could trust may have led to a quicker diagnosis. The majority of respondents (82%; *n* = 281) agreed that the financial cost of care and treatment was a worry at diagnosis; this varied according to race/ethnicity, with 95% (*n* = 83) of non-Hispanic Black, 76% (*n* = 57) of Hispanic, and 77% (*n* = 122) of non-Hispanic White respondents agreeing.

**Figure 1.**
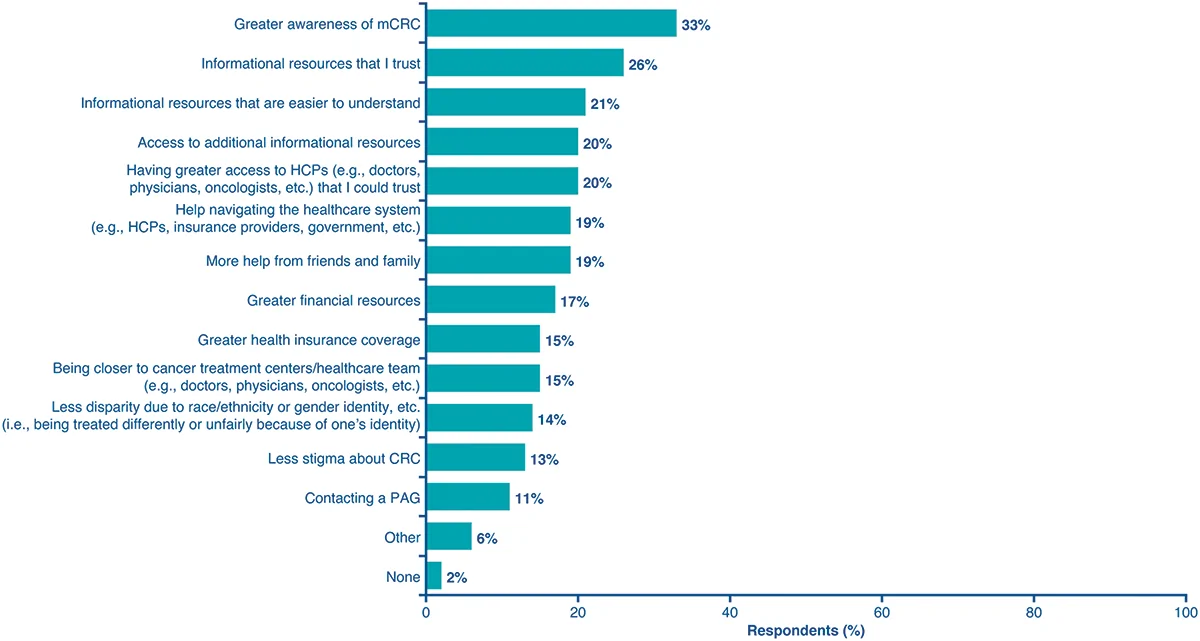
Factors that respondents felt could have led to a quicker diagnosis*.

*Respondents could select more than one response.

CRC, colorectal cancer; HCP, health-care provider; mCRC, metastatic colorectal cancer; PAG, patient advocacy group.

### 3.4. Information and support needs

At diagnosis, 83% (*n* = 286) of respondents wished that more information was provided to them and 43% (*n* = 148) felt they did not know where they could go to connect with others diagnosed with mCRC about their disease and experience (Supplementary Figure S2). Conversely, in response to a separate question, 74% (*n* = 256) of respondents also stated that they felt overwhelmed by the information that was provided by their health-care provider (HCP) at diagnosis. Overall, 46% (*n* = 157) reported being “very” or “somewhat familiar” with mCRC-related PAGs, including 38% (*n* = 33) of non-Hispanic Black, 47% (*n* = 35) of Hispanic, and 50% (*n* = 79) of non-Hispanic White respondents. During treatment, 19% (*n* = 64) wished they had more support from PAGs, and 17% (*n* = 59) stated that they had relied on PAGs to help guide their treatment decisions.

### 3.5. Impact on QoL

Overall, respondents felt that mCRC had a negative impact on their QoL, including being able to maintain a “normal” work schedule, financial well-being, and emotional well-being/mental health (Figure 2). Over two-thirds of respondents (69%; *n* = 223) felt it was difficult to access resources to help with balancing mCRC and their overall life, and 65% (*n* = 209) found it difficult to balance mCRC with their family responsibilities; this included 59% (*n* = 111) of males and 72% (*n* = 98) of females. In terms of the impact on relationships, 72% (*n* = 247) felt they had been a burden to their family and 63% (*n* = 216) felt the support they received from friends/family had decreased over the course of the mCRC experience.

**Figure 2.**
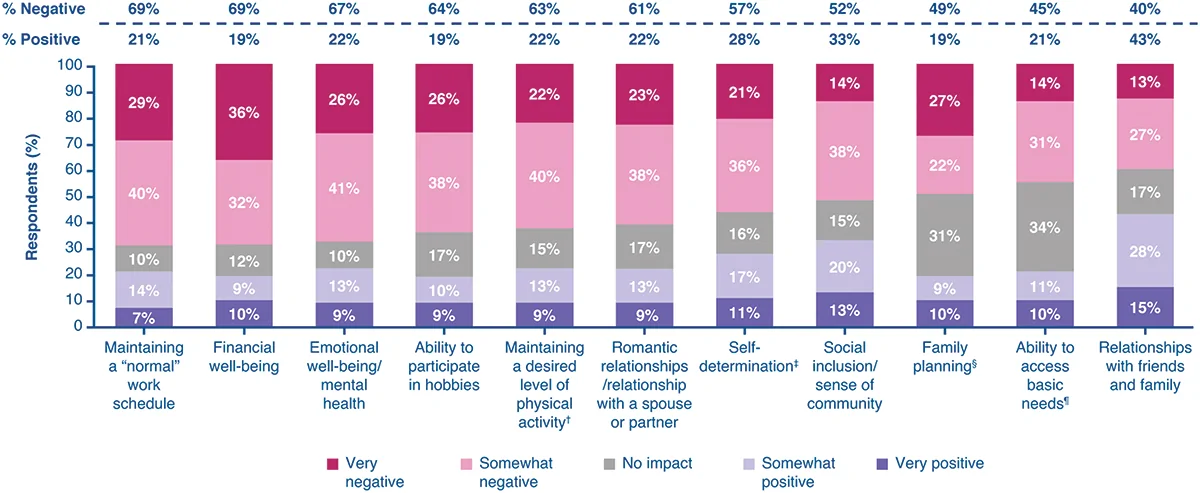
Factors that respondents felt impacted their QoL*.

*Totals may not sum to 100% due to rounding; excludes “not applicable” responses.^†^E.g., eating, dressing, etc. and chores (e.g., washing dishes, taking out the trash, etc.).^‡^I.e., having a sense of control over one’s life^.§^E.g., safe housing, food, transportation, etc.^¶^E.g., infertility issues, fertility preservation, delaying having children or deciding not to have children, etc.

QoL, quality of life.

Of note, there was also a positive impact following the mCRC diagnosis with 70% (*n* = 241) of respondents agreeing that their diagnosis inspired them to take action on things they had always wanted to do or actions that they had pushed off, and 64% (*n* = 219) agreeing that living with mCRC had helped them to better enjoy the little things in life (Supplementary Figure S2).

#### 3.5.1. Mental health impacts

Many respondents felt that a diagnosis of mCRC had a high emotional impact on them, with 78% (*n* = 270) agreeing that, since diagnosis, they have felt alone in their experience. Almost all respondents reported that they had tried to access mental health resources (96%; *n* = 330); however, 53% (*n* = 183) felt that they experienced difficulties accessing these resources (Supplementary Figure S2), including 39% (*n* = 29) of Hispanic, 54% (*n* = 85) of non-Hispanic White, and 62% (*n* = 54) of non-Hispanic Black respondents.

#### 3.5.2. Financial impacts

Most respondents (88%; *n* = 302) reported a negative financial impact of their mCRC diagnosis and treatment; this included 80% (*n* = 127) of non-Hispanic White, 92% (*n* = 80) of non-Hispanic Black, and 97% (*n* = 73) of Hispanic respondents, with a decrease in income affecting 47% (*n* = 160) of respondents (Figure 3). Furthermore, 63% (*n* = 218) of respondents agreed that having limited resources available to meet basic needs during treatment (e.g., safe housing, food, and transportation) was a burden to them and their family (Supplementary Figure S2); this included 53% (*n* = 83) of non-Hispanic White, 75% (*n* = 56) of Hispanic, and 77% (*n* = 67) of non-Hispanic Black respondents. Respondents felt that resources to support economic hardship would help to improve their mental health/decrease stress (48%; *n* = 166) and foster hope for the future (42%; *n* = 145).

**Figure 3.**
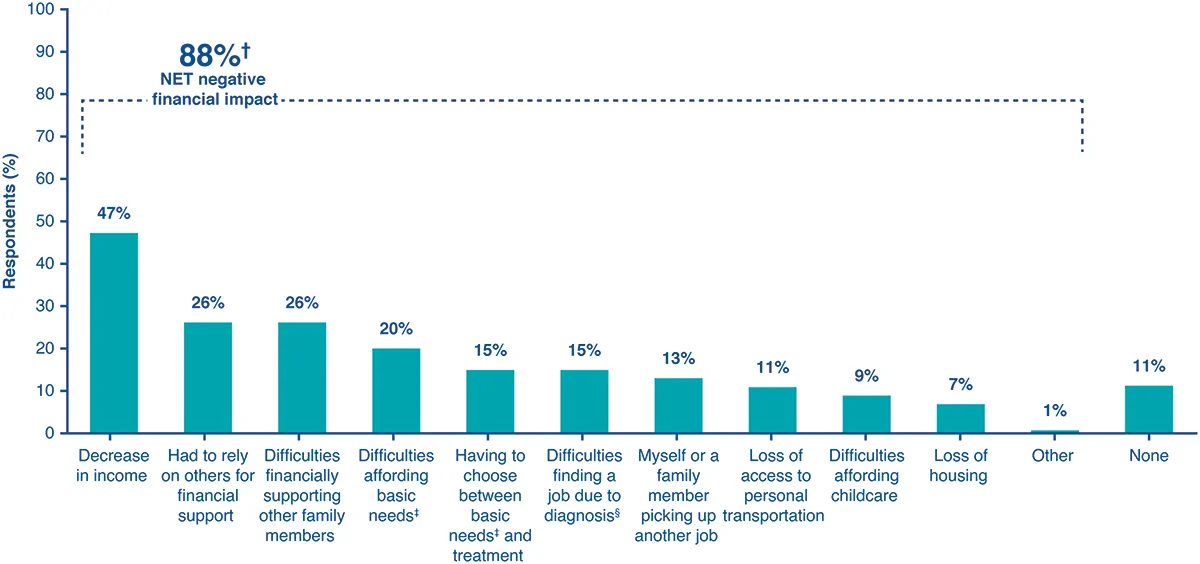
Financial impacts that respondents reported experiencing as a result of mCRC treatment and diagnosis*.

*Respondents could select more than one response.^†^Net percentage accounts for respondents who selected multiple responses.^‡^E.g., safe housing, food, transportation, etc.^§^E.g., treatment schedule, accommodations available, symptoms, etc.

mCRC, metastatic colorectal cancer.

### 3.6. Access to care

Overall, 39% (*n* = 135) of respondents reported that they found it difficult to receive the necessary medical care and treatments for mCRC; this included 29% (*n* = 22) of Hispanic, 39% (*n* = 62) of non-Hispanic White, and 49% (*n* = 43) of non-Hispanic Black respondents. In addition, 47% (*n* = 73) of females and 33% of males (*n* = 62) reported having difficulty receiving necessary care, including finding HCPs, scheduling appointments, getting medical guidance, and making decisions. Respondents felt that a range of factors negatively impacted their ability to receive necessary care, primarily financial limitations for 35% (*n* = 120) (Supplementary Figure S3), with 24% (*n* = 18) of Hispanic, 32% (*n* = 50) of non-Hispanic White, and 48% (*n* = 42) of non-Hispanic Black respondents reporting that they experienced this.

### 3.7. Culturally competent care and health inequities

In total, 72% of the respondents (*n* = 247) felt that their HCP incorporated their cultural background into their treatment and care plans “very well” or “somewhat well”; this included 60% (*n* = 52) of non-Hispanic Black, 75% (*n* = 56) of Hispanic, and 75% (*n* = 118) of non-Hispanic White respondents. However, in response to a separate question, 20% of the respondents (*n* = 68) reported that their HCP and their overall health-care team did not understand (“not very well” or “not at all well”) how their cultural background influenced their mCRC experience, including 15% (*n* = 11) of Hispanic, 15% (*n* = 23) of non-Hispanic White, and 34% (*n* = 30) of non-Hispanic Black respondents. Additionally, 57% (*n* = 195) felt unheard or dismissed at least sometimes during a healthcare experience related to mCRC, including 64% (*n* = 68) of respondents aged ≥65 years, and 52% (*n* = 82) of non-Hispanic White, 54% (*n* = 47) of non-Hispanic Black, and 67% (*n* = 50) of Hispanic respondents. The main ways in which respondents felt that they could be heard, seen, and valued when interacting with their mCRC care team included having more conversations about their mCRC outside of their medical experience (e.g., about managing daily activities, QoL, and ability to work); having more conversations about the financial difficulties they experienced due to their diagnosis and being acknowledged when they had concerns with their care plan (Supplementary Figure S4). Furthermore, 74% (*n* = 254) reported that aspects of their mCRC experience were negatively impacted by health inequities that they have faced (Figure 4); this included 55% (*n* = 87) of non-Hispanic White, 91% (*n* = 79) of non-Hispanic Black, and 92% (*n* = 69) of Hispanic respondents. In addition, 49% (*n* = 167) reported that health inequities had negatively impacted their care (quality, timing, or access). In addition, 49% (*n* = 136) of the applicable respondents felt that mCRC had a negative impact on family planning (e.g., infertility issues, fertility preservation, delaying having children or deciding not to have children, etc.), including 61% (*n* = 68) of females and 41% (*n* = 68) of males for whom this is applicable.

**Figure 4.**
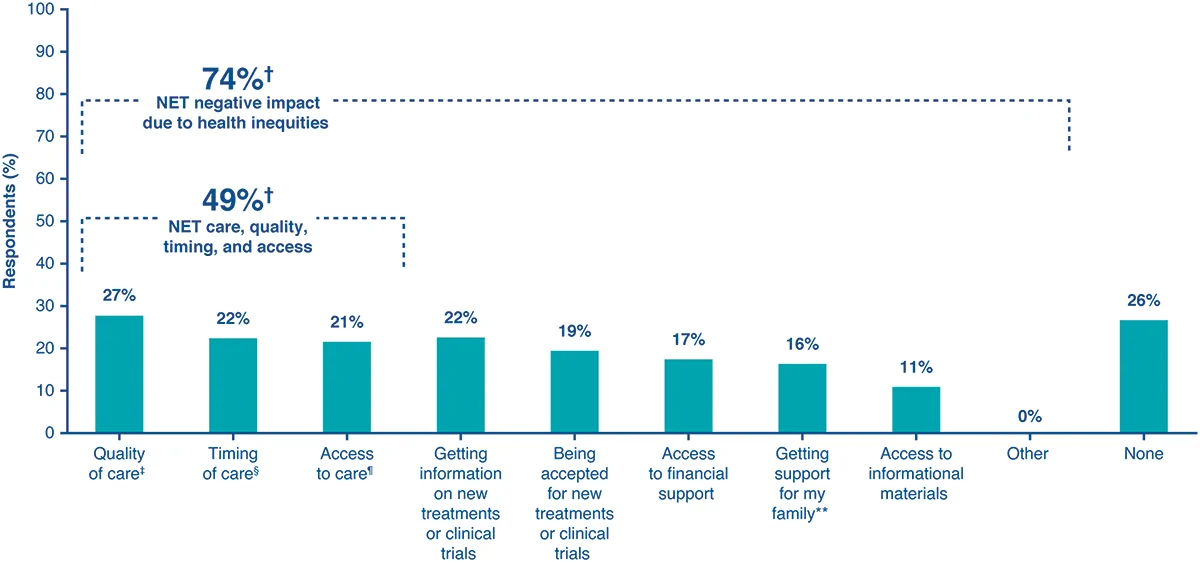
Aspects of living with mCRC that respondents reported were negatively impacted by the health inequities they faced*.

*Respondents could select more than one response.^†^Net percentage accounts for respondents who selected multiple responses.^‡^E.g., being rushed, not feeling listened to, not being offered treatments/care that works for you, etc.^§^E.g., screenings being delayed, treatments and access to results not being prioritized, etc.^¶^E.g., ability to travel to appointments, ability to afford treatments, not being given the option of screening or treatments, or resources to support access to treatments, etc. **, E.g.,, care taking information/resources, hotel rooms during treatment, etc.

mCRC, metastatic colorectal cancer.

### 3.8. Treatment decisions

Overall, 74% (*n* = 256) of respondents reported that they had received treatment for their mCRC; this included 57% (*n* = 60) aged ≥65 years, and 60% (*n* = 52) of non-Hispanic Black, 80% (*n* = 60) of Hispanic, and 82% (*n* = 129) of non-Hispanic White respondents. Among those who had received treatment, 41% (*n* = 142) reported being in active treatment at the time of completing the survey, including 26% (*n* = 28) aged ≥65 years. Following diagnosis with mCRC, 9% (*n* = 7) of respondents who were ≤44 years of age, 8% (*n* = 13) between 45 and 64 years of age, and 24% (*n* = 25) who were ≥65 years of age reported that they had decided not to receive any treatment. When making treatment decisions, 87% (*n* = 298) reported relying on HCPs, primarily oncology surgeons (48%, *n* = 166) or oncologists (45%, *n* = 154). In addition, 36% (*n* = 123) reported using internet searches to guide decisions relating to their treatment and 78% (*n* = 268) often independently researched new treatments/clinical trial options. Factors that respondents felt could improve their treatment experience included emotional support services (43%, *n* = 129), a better understanding of treatment options (39%, *n* = 116), and a better understanding of disease progression (34%, *n* = 102). In terms of involvement in the treatment decision-making process, 67% (*n* = 231) of respondents wished they had more power over decisions made about their treatment (Supplementary Figure S2); this included 80% (*n* = 85) of those aged ≥65 years (versus 61% [*n* = 146] aged <65 years), and 56% (*n* = 89) of non-Hispanic White, 68% (*n* = 51) of Hispanic, and 83% (*n* = 72) of non-Hispanic Black respondents. The main factors that respondents felt affected treatment decisions were the impact on their physical health, the QoL implications, and the fear of being a burden to their family (Figure 5). Furthermore, 13% (*n* = 44) of respondents said that family planning had impacted their mCRC treatment decisions, including 23% (*n* = 18) of those aged ≤44 years, 11% (*n* = 18) of those aged between 45 and 64 years, and 8% (*n* = 8) of those aged ≥65 years.

**Figure 5.**
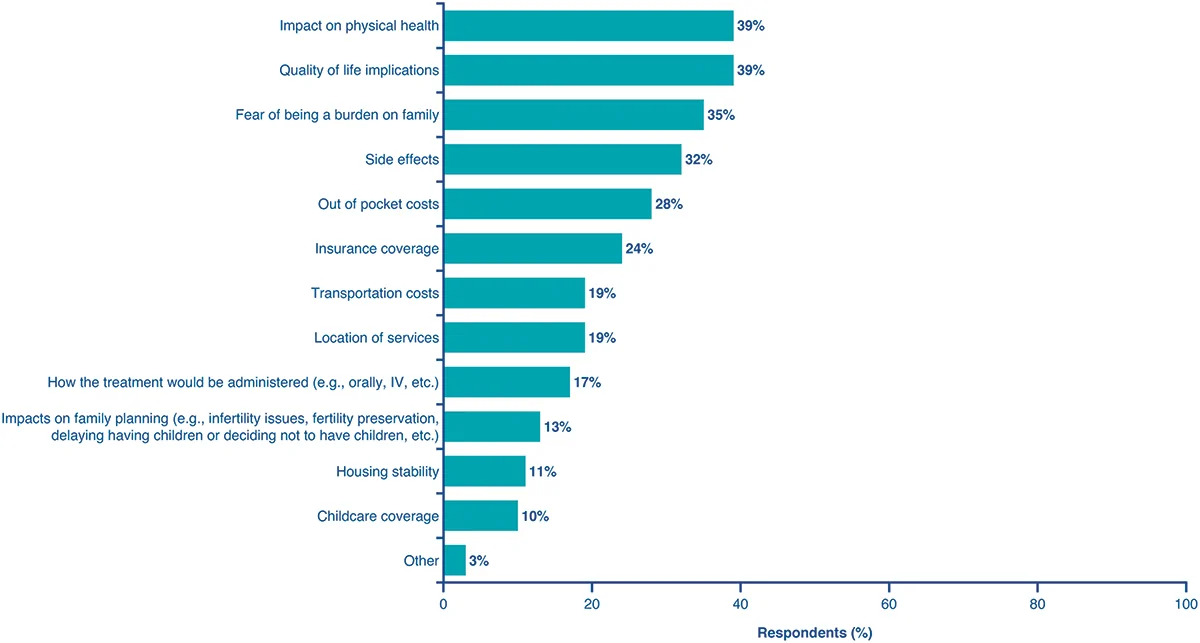
Factors that respondents felt impacted mCRC treatment decisions*.

*Respondents could select more than one response.

IV, intravenously; mCRC, metastatic colorectal cancer.

Overall, 67% (*n* = 172) of respondents agreed that outside conditions, such as finances and mental health, impacted the ability to take treatment as recommended; this included 59% (*n* = 76) of non-Hispanic White, 72% (*n* = 43) of Hispanic, and 79% (*n* = 41) of non-Hispanic Black respondents.

Furthermore, 36% (*n* = 92) reported that they had stopped taking/receiving treatments due to the overall burden (e.g., due to the financial impact, QoL, etc.), including 21% (*n* = 27) of non-Hispanic White, 54% (*n* = 28) of non-Hispanic Black, and 55% (*n* = 33) of Hispanic respondents. Of respondents aged ≥65 years and <65 years, 42% (*n* = 25) and 34% (*n* = 67) reported that they had stopped taking/receiving treatments due to the overall burden, respectively. In total, 39% (*n* = 134) reported that QoL had implications on the decisions they had to make regarding their mCRC treatment.

## 4. Discussion

Publications reporting patient perceptions relating to their diagnosis, treatment, and experience of living with mCRC are limited [19]. The results of this survey enhance our understanding of the challenges patients with mCRC face and provide insights into the information and support needs that patients feel would improve their healthcare experience. Important gaps were highlighted in the diagnosis experience, information and support needs, impact on QoL, access to care, culturally competent care, and patient involvement in treatment decision-making.

Respondents of our survey were generally representative of the mCRC patient population in the US, including by minority ethnic group. For example, 6% of the respondents identified as Asian or from the Pacific Islands and 1% as American Indian or as Alaska Native, consistent with national CRC incidence patterns (of all patients diagnosed with CRC in the US, approximately 4 − 11% are Asian or from the Pacific Islands; and 1% are American Indian or Alaska Native) [20,21]. However, we note that the mean age of respondents for this survey (56 years) was lower than the median age identified among patients diagnosed with mCRC between 2010 and 2019 in the US (71 years) [22]. In addition, the reported household income for 74% of the survey respondents was $75,000 or higher (including 51% who reported $100,000–$199,999 and 6% who reported $200,000 or more), which is greater than the general US population in 2022 (median $74,580) [23]. However, 47% of the respondents’ highest level of education was a 4-year college degree or higher, which was consistent with the overall proportion of the US population in 2022 (43%) [24].

The survey findings showed that around one-third of respondents with mCRC felt that they experienced a delay in diagnosis, which occurred more frequently in females (38%) and respondents ≤44 years of age (47%); this perception of a delay in diagnosis in females and young patients is consistent with previous findings in patients with CRC [25]. For females, symptoms associated with CRC, such as constipation and abdominal pain, may be misdiagnosed as irritable bowel syndrome or urinary tract infections, both of which are more common in females than males [25,26]. Similarly, a delay in diagnosis of CRC among young patients may be attributed to prolonged time to presentation or misdiagnosis [27]. Practical solutions to address the disparities that may contribute to diagnostic delays are important, for example, recommending colonoscopies or rectal exams for patients who experience a change in bowel movements and may not qualify for routine screening [28].

Improving access to appropriate information and disease awareness were identified by respondents as important factors that could have led to a faster diagnosis. Most respondents (83%) wished that their HCP had provided more information at the time of initial diagnosis; however, conversely, 74% felt overwhelmed by the information provided by their HCP when first diagnosed. It has been shown previously that experiences relating to health information needs for cancer were negative when an overwhelming amount of information was received [29]. Further studies into distinguishing essential information from superfluous material will be critical in preventing patients with mCRC from feeling overwhelmed while ensuring appropriate levels of information provision and education. In addition, levels of health literacy in older adults, as well as Hispanic (especially those who exclusively speak Spanish) and Black individuals are often lower than younger adults or White individuals [30–32], which presents barriers for these groups in being able to access information and highlights the importance of providing easy-to-understand and culturally responsive information, not only at initial diagnosis but throughout the mCRC treatment journey.

The role of PAGs is important to help improve the experience of patients with cancer, through raising awareness, providing education on disease and treatment options, providing informational resources and psychological support services to address various medical and non-medical needs, informing policy decisions, and representing patients to ensure their voices are heard [33]. Familiarity with PAGs and accessing the support available can positively impact patient expectations, diagnosis, and treatment [34]. However, we found that only 46% of the respondents reported being “very” or “somewhat familiar” with PAGs, which was notably lower among non-Hispanic Black respondents (38%), compared with Hispanic (47%), and non-Hispanic White (50%) respondents. There is a need to increase awareness of PAGs to enhance visibility of the support they can provide. Collaborations between PAGs may be an effective approach to develop tailored resources that meet specific patient needs. For example, COLONTOWN and BLKHLTH partnered on a series of events to identify opportunities to improve awareness of, access to, and engagement with psycho-social support for Black patients with CRC.

Overall, we found that mCRC had a strongly negative impact on QoL, with the majority of respondents being “somewhat” or “very” negatively impacted across multiple factors contributing to QoL, suggesting that there is an opportunity to substantially improve the patient experience. QoL in patients with mCRC can be impacted by a range of factors, including patient characteristics, socioeconomic factors, disease-related factors, and treatment-related effects [35]. Respondents in our survey felt that financial concerns and emotional well-being had the greatest negative impact on their QoL. Respondents also reported their mCRC diagnosis having a positive impact on their outlook and mind-set. Support from friends and family has been shown to be beneficial for patients with cancer [36]; however, we found that around two-thirds of respondents felt the support received from friends and family decreased over the course of their disease. This unmet medical need can be addressed by PAGs and significantly improve patients' experience with their disease.

We found that 72% of females reported difficulty in accessing resources to help with balancing mCRC and their family responsibilities, compared with 59% of males. Nearly all respondents reported that they had attempted to access mental health resources; however, around half who tried reported having difficulty with access, increasing to nearly two-thirds for non-Hispanic Black patients. A meta-analysis of 183 studies across different cancer types found that depression was most prevalent among patients with CRC, due to the high mortality rate and physical disabilities associated with the disease [37]. In addition, while patients of all races and ethnicities report impact of CRC on mental health, Hispanic and Black patients with CRC are more likely to report fair or poor mental health status compared with White patients [38]. Therefore, ensuring mental health support and resources are accessible is a key unmet need.

The financial burden of the mCRC was a major concern among respondents, with 88% reporting that they had experienced a financial impact; this included 92% of non-Hispanic Black and 97% of Hispanic respondents, compared with 80% of non-Hispanic White respondents. One of the first studies to evaluate financial hardship among those living with mCRC reported that nearly 75% of the patients experienced major financial hardship within 1 year of diagnosis [39]. Those from Black and minority racial/ethnic backgrounds are over two times more likely to be financially impacted by a cancer diagnosis and over five times more likely to be denied insurance than White patients [40,41]. In our survey, almost 40% of the respondents reported experiencing difficulty receiving necessary care for their mCRC, with financial limitations cited as a barrier by more than one-third. Respondents identified financial assistance as being an important need for informational resources and felt resources aimed at economic hardship would help reduce stress and give them hope for the future. It is important for HCPs to be aware of the financial challenges their patients face and to help mitigate the financial challenges by discussing treatment costs and signposting patients to resources that can support them with financial hardship. Indeed, 70% of the adults in the US, across multiple health conditions, want to have conversations about the costs of care with their HCPs, but only 28% report doing so [42]. Novel programs to improve cost-health literacy during physician training can overcome this disparity [43].

Despite efforts to encourage and improve cultural competence and patient-centered approaches in routine clinical care [44], our survey found that 57% of the respondents reported feeling unheard or dismissed by their HCP at least sometimes. Furthermore, 74% reported that aspects of their mCRC experience were negatively impacted by health inequities, including 91% of non-Hispanic Black and 92% of Hispanic respondents, compared with 55% of non-Hispanic White respondents. Our findings are consistent with a Canadian patient survey, which found that 39% of patients with CRC felt dismissed by their HCP [45]. A higher proportion of non-Hispanic Black respondents reported having stage 1 CRC at the time of diagnosis compared with Hispanic or non-Hispanic White respondents, suggesting that non-Hispanic Black respondents may have received quicker or more timely diagnoses compared with non-Hispanic White or Hispanic respondents. However, this did not change their perception of health disparities, consistent with previous research in relation to trust in the healthcare system [46]. HCPs play a key role in reducing disparities in patient care; having more conversations about medical, non-medical, and financial difficulties can help patients feel heard, seen, and valued. In addition, tailoring patient interactions according to their individual needs, concerns, and cancer stage may help to improve the patient experience and outcomes. While we recognize that there is a common disparity between patient perceptions of how their concerns are received by HCPs [45] and how HCPs believe they communicate with patients [47], the survey was not designed to determine whether this issue reflects an objective problem or a subjective perception.

Shared decision-making between patients and HCPs regarding treatment is considered best practice [48,49]; however, our survey revealed that 67% of the respondents wished they had more power in making decisions regarding their treatment, including 80% of those aged ≥65 years and 83% of non-Hispanic Black respondents. Furthermore, we found that a higher proportion of respondents aged ≥65 years reported foregoing treatment following diagnosis with mCRC compared with younger respondents. Incorporation of patient values and preferences in cancer treatment decisions is important as older patients’ preferences (e.g., preservation of QoL) may differ from those of younger patients who may be more willing to tolerate potential adverse effects of treatment to extend life expectancy [50]. By having more power in the treatment planning process, older patients may feel more optimistic about the future. Previous research has shown that race, ethnicity, and language are significant factors associated with active participation in care; for example, Black patients tend to receive less detailed information on treatment from HCPs versus White patients, and therefore it may be more difficult for Black patients to make informed decisions about their treatment [51,52]. Treatment programs that focus on shared decision-making represent a key area to help advance patient care and ensure that patients from all backgrounds feel heard and patient outcomes are optimized.

### 4.1. Limitations

All surveys and polls, whether they use probability sampling or not, are subject to multiple sources of error, which are most often not possible to quantify or estimate, including, but not limited to, coverage errors, errors associated with non-response, errors associated with question wording and response options, and post-survey weighting and adjustments. Furthermore, the raw data were not weighted and are therefore only representative of the individuals who completed the survey. Statistical comparison was descriptive only, and formal statistical analysis was not conducted. Due to the nature of the research, it was not possible to validate responses using patient records, including verification of self-reported mCRC diagnoses. As respondents recruited through PAGs actively engaged with and sought support from PAGs, they may not be representative of the general mCRC population. In addition, the ability of respondents to accurately recall information may have been a source of bias. Analysis by race/ethnicity did not include Asian respondents, those who identified as native Hawaiians or from the Pacific Islands, or American Indian or Alaska Native respondents due to small patient numbers, as they represented only 6% of the total respondents. Finally, the survey was conducted in English, which may have affected participation rates.

## 5. Conclusions

The findings of this survey identified several gaps in care that may be detrimental to the experiences and outcomes of patients with mCRC; these include a lack of information provision, support, access to mental health resources, understanding of financial concerns, and patient involvement in decision-making. In addition, we found that many patients reported being unfamiliar with PAGs, despite their important role in providing support to patient and caregivers throughout the cancer journey. Collaboration between PAGs, patients, caregivers, oncology social workers, patient navigators, and the multidisciplinary healthcare team may help improve understanding of the diverse needs of people with mCRC, signposting to key information and socio-emotional resources to provide timely support beginning at diagnosis, and help alleviate concerns and empower patients to take an active role in their mCRC management.

Further research is needed to better understand informational needs and patient preferences across racial/ethnic groups around accessibility to health-care services and resources, with a focus on mental health and financial resources.

## Supplementary Material

Supplemental Material

Supplemental Material

CROSS checklist.docx

Newcomer mCRC patient survey supplement.docx

Newcomer_Infographic_9Jun26.pdf

## Data Availability

The datasets generated during and/or analyzed during the current study are available from the corresponding author on reasonable request.

## Article highlights

- To continue to improve quality of life and optimize outcomes for patients with metastatic colorectal cancer (mCRC), it is critical to gain insights into the experiences and challenges they face.
- We conducted an online survey involving 344 respondents in the United States (US) to evaluate patient perspectives on their care and treatment experiences.
- Overall, 31% of the respondents reported that their mCRC diagnosis was delayed, and 83% wished that more information had been provided by their health-care provider at diagnosis.
- Mental health resources were difficult to access for 53% of the respondents; 88% were impacted by the financial burden of mCRC; 49% reported health inequities affecting the quality and timing of their care, and their access to care; and 67% wished for more power in the treatment decision-making process.
- Results of this survey highlight multiple gaps in care for patients with mCRC in the US which are amplified in certain subgroups and may be detrimental to the experiences and outcomes of patients.
- To address these gaps, a better understanding of informational needs and a focus on shared decision-making is required.
