# Supplementary material for: Patient experiences and unmet needs in diagnosis and treatment of metastatic colorectal cancer: results of a US survey
Source: Future Oncol. 2026 Oct 10;22(20):2445–56. doi: 10.1080/14796694.2026.2695213 (PMC13685566; doi:10.1080/14796694.2026.2695213)
Supplement: Supplementary file 1 [file IFON_A_2695213_SM8119.pdf]

Patient experiences and unmet needs in diagnosis and treatment of metastatic colorectal cancer: results of a US survey

Background

- Symptoms of mCRC can impair patients' physical, social, and psychological well being
- Incidence and survival of mCRC vary substantially depending on patient characteristics
- We conducted an online patient survey to better understand the perspectives of patients living with mCRC

Methods

Participants

Patients residing in the US aged ≥18 years with mCRC

344 respondents

- **254 recruited via online panels**
- **90 recruited via PAGs** (BLKHLTH, COLONTOWN, Colorectal Cancer Alliance, and Family Reach)

Survey design

Online 60-question survey, conducted by The Harris Poll (January 17–February 7, 2024)

Scope of questions covered

Patient profile, diagnosis experience, the role of PAGs/other resources, QoL, access to care, financial challenges, health inequities, and treatment experience

Key Demographics

- **Mean (SD) age:** 56 (±13) years
- **Males:** 55% (n=190)
- **Females:** 45% (n=154)
- **Non-Hispanic White:** 46% (n=158)
- **Non-Hispanic Black/African American:** 25% (n=87)
- **Hispanic:** 22% (n=75)

Results

31% of respondents (n=105/344) felt their mCRC diagnosis was delayed

Common factors that respondents felt could have led to a quicker diagnosis:*

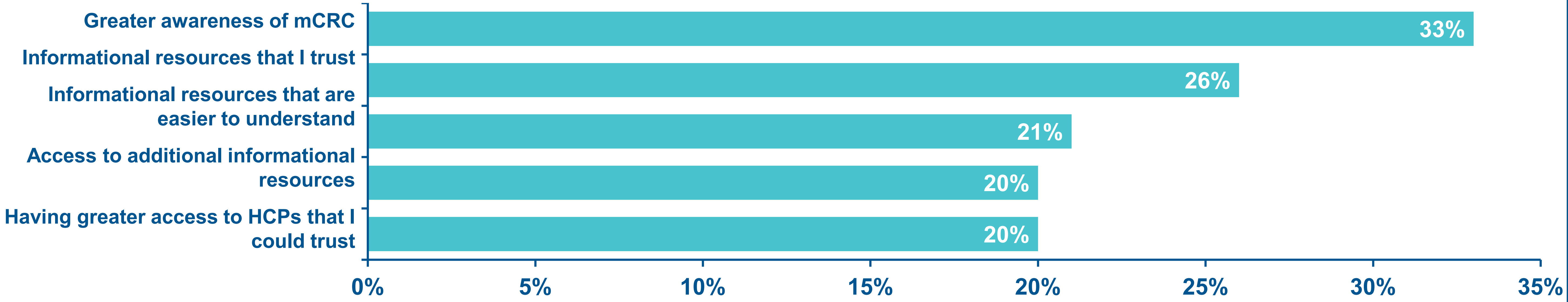

83% of respondents (n=286/344) wished for more information at diagnosis

'I wish I had more information provided to me at diagnosis…'

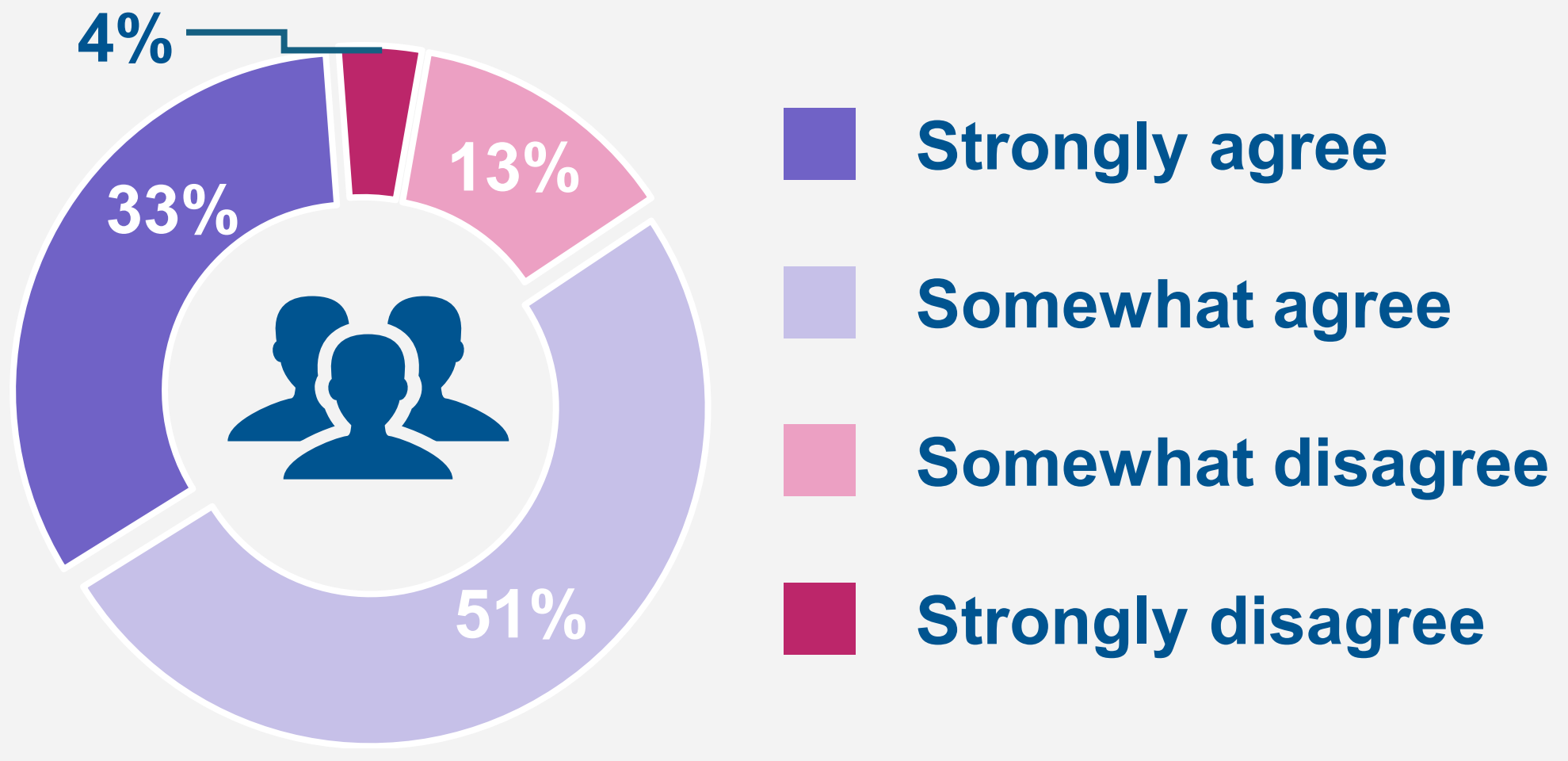

53% of respondents (n=183/344) found it difficult to access mental health resources

'How easy or difficult has it been to access necessary resources to help with mental health?'

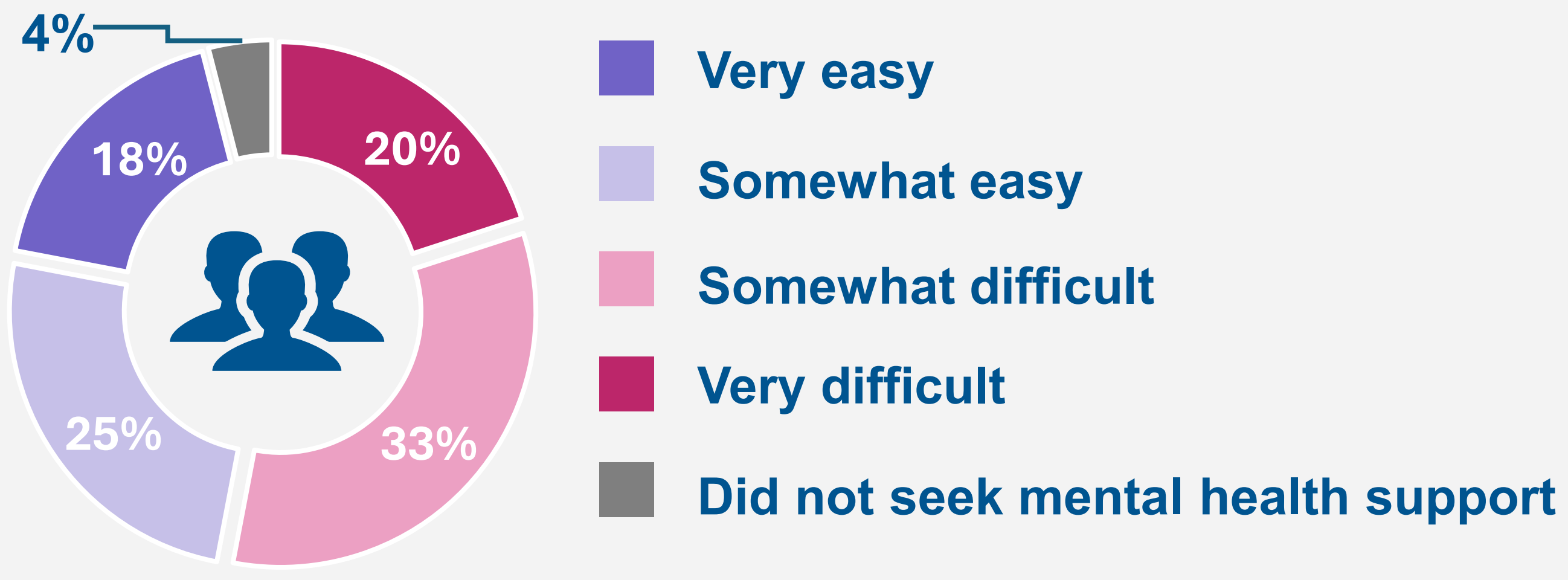

67% of respondents (n=231/344) wished for more power in treatment decision-making

'I wish I had more power in making treatment decisions…'

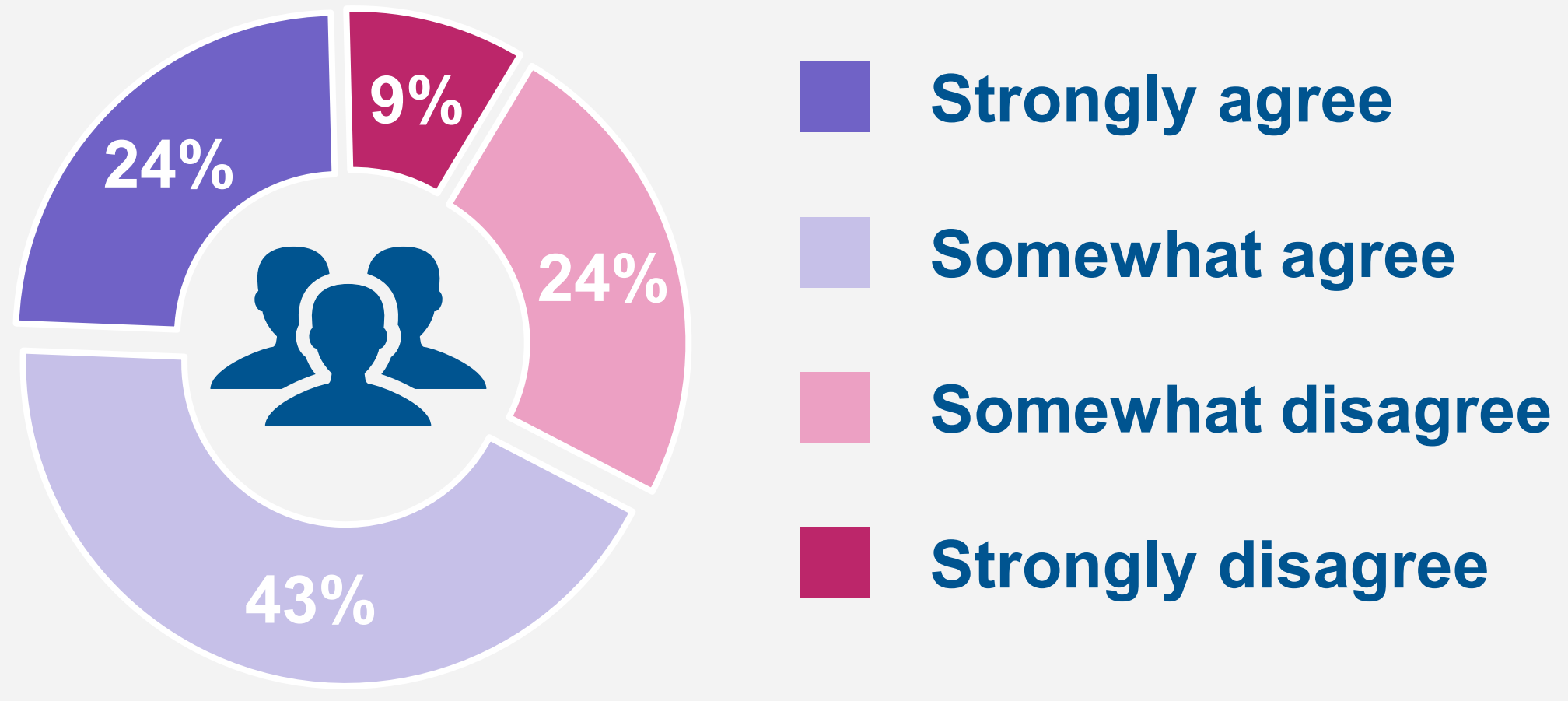

88% of respondents (n=302/344) reported a negative financial impact of mCRC

Common financial impacts reported by respondents due to mCRC:*

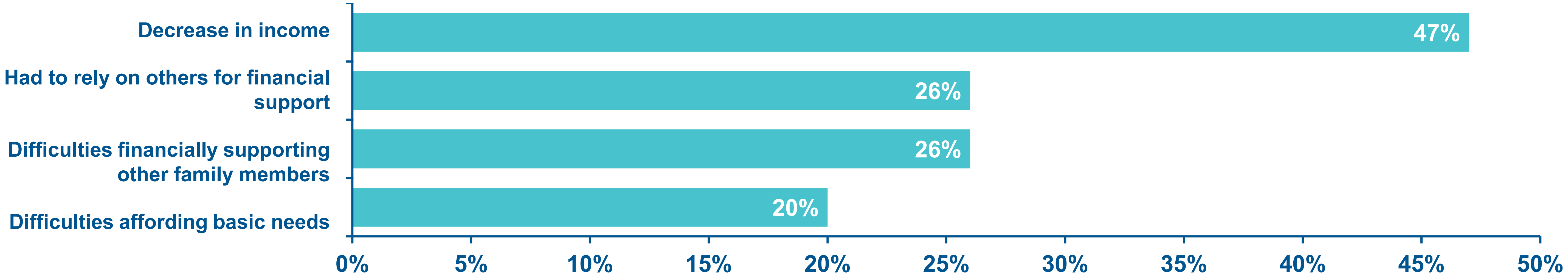

Conclusions

The survey highlights key unmet patient needs in mCRC care. Access to informational resources, including for financial assistance, and a greater emphasis on shared treatment decision-making would help patients to feel valued and improve their mCRC experience.

*Reported by ≥20% of respondents; respondents could select more than one response. HCP, healthcare professional; mCRC, metastatic colorectal cancer; PAGs, patient advocacy group; QoL, quality of life; SD, standard deviation; US, United States.
