# Supplementary material for: Patient experiences and unmet needs in diagnosis and treatment of metastatic colorectal cancer: results of a US survey
Source: Future Oncol. 2026 Oct 10;22(20):2445–56. doi: 10.1080/14796694.2026.2695213 (PMC13685566; doi:10.1080/14796694.2026.2695213)
Supplement: Supplementary file 2 [file IFON_A_2695213_SM1456.docx]

**Patient experiences and unmet needs in diagnosis and treatment of metastatic colorectal cancer: results of a US survey**

**Video Abstract Transcript**

Hello, my name is Kim Newcomer and I’m a stage IV colorectal cancer survivor of 18 years, and I’m with the Colorectal Cancer Alliance, a patient advocacy organization based in Washington DC. On behalf of my coauthors, I’m pleased to present the findings from a US survey examining patient experiences and unmet needs in metastatic colorectal cancer. This work reflects what patients tell us they need most, beyond clinical outcomes.

Colorectal cancer is the third most commonly diagnosed cancer, globally. At diagnosis, approximately 20% of patients present with metastatic disease, and up to half of those initially diagnosed without metastases will develop metastatic disease over time. Although survival outcomes have improved over the last 30 years, disparities in incidence, mortality, and care persist, particularly across racial and ethnic groups. Patients will also experience high symptom burden, including nausea, fatigue, gastrointestinal symptoms, and pain, which can significantly affect physical, emotional, and social wellbeing.

To better understand patient challenges and opportunities for improvement, we conducted a national survey of people living with metastatic colorectal cancer in the US. To improve care, we first need to understand the patient perspective. The survey was conducted by the Harris Poll, in collaboration with leading patient advocacy groups:

- BLKHLTH
- COLONTOWN
- The Colorectal Cancer Alliance
- And Family Reach

Participants were aged eighteen or older with self-reported diagnosis of stage 4 metastatic colorectal cancer. The survey comprised 60 questions in English which explored patient profile, diagnosis experience, the role of patient advocacy groups and other resources, quality of life, access to care and financial challenges, health inequities, and treatment experience. In total, 344 eligible respondents completed the survey, of which 254 were recruited via online panels, and 90 were recruited via patient advocacy groups. The mean age of respondents was 56, and 55% were male. 46% of the respondents identified as non-Hispanic White; 25% as non-Hispanic Black, or African American, and 22% as Hispanic. To improve care, we first need to understand the patient perspective.

Nearly one third of respondents felt like their metastatic colorectal cancer diagnosis had been delayed. Patients reported that earlier diagnosis could have been supported by a greater general awareness of colorectal cancer, access to reliable information, and access to clear and easy to read educational resources. Additionally, 83% percent wished they had received more information at the time of their diagnosis, and 43% did not know where to connect with other patients who shared their experience.

Respondents reported that metastatic colorectal cancer had a substantial negative impact on their quality of life. Key affected areas included maintaining employment, financial stability, and emotional or mental, wellbeing. While 96% reported attempting to access mental health support, more than half experienced difficulty when obtaining these services.

Most respondents reported a negative financial impact related to their diagnosis and treatment. Now, nearly half experienced a reduction in income, and approximately one quarter reported relying on others for financial support, or had difficulties supporting their families. Additionally, 63% reported that limited access to basic needs, including housing, food, and transportation, created a burden during treatment.

Nearly three quarters of respondents reported that health inequities negatively affected their cancer experience. Almost half reported impacts of quality, timing, or accessibility of their care. In shared decision-making, 67% expressed a desire for greater involvement in their treatment decisions. Patients want to be partners in their care, not passive recipients.

Understanding the lived experience of patients with metastatic colorectal cancer is essential to improving the quality of life and clinical outcomes. This study found that many patients perceive diagnostic delays, experience emotional and financial hardship, and feel the impact of mental health inequities in their care. These findings highlight meaningful gaps in information access, psychosocial support, financial resources, and shared decision-making. Addressing these gaps will require stronger patient education, improved care navigation, expanded mental health and social support, and more a patient-centred approach to treatment planning.

Listening to patients is one of the most powerful tools we have to improving cancer care. Our hope is that these insights lead to more equitable, informed, and companionate care for people living with metastatic colorectal cancer.
