# Supplementary material for: Patient experiences and unmet needs in diagnosis and treatment of metastatic colorectal cancer: results of a US survey
Source: Future Oncol. 2026 Oct 10;22(20):2445–56. doi: 10.1080/14796694.2026.2695213 (PMC13685566; doi:10.1080/14796694.2026.2695213)
Supplement: Supplementary file 3 [file IFON_A_2695213_SM8121.docx]

**SUPPLEMENTARY MATERIAL**

**Patient experiences and unmet needs in diagnosis and treatment of metastatic colorectal cancer: results of a US survey**

**Supplementary Table 1. Patient circumstances and logistical information.**

| **Characteristic** | **All respondents**  **(*N* = 344)** | **Non-Hispanic Black/African American respondents**  **(*n* = 87)*** | **Non-Hispanic  White respondents**  **(*n* = 158)** | **Hispanic respondents**  **(*n* = 75)***^†^ |
| --- | --- | --- | --- | --- |
| **Urbanicity, *n* (%)** | |  |  |  |
| Urban or city area | 234 (68) | 67 (77) | 87 (55) | 59 (79) |
| Suburban area next to city | 85 (25) | 18 (21) | 50 (32) | 15 (20) |
| Small town or rural area | 25 (7) | 2 (2) | 21 (13) | 1 (1) |
| **Household income, *n* (%)** | | |  |  |
| LESS THAN $35,000 (NET) | 26 (8) | 7 (8) | 13 (8) | 6 (8) |
| Less than $15,000 | 6 (2) | 4 (5) | 2 (1) | 0 |
| $15,000 to $24,999 | 9 (3) | 2 (2) | 5 (3) | 2 (3) |
| $25,000 to $34,999 | 11 (3) | 1 (1) | 6 (4) | 4 (5) |
|  |  |  |  |  |
| $35,000 to $49,999 | 22 (6) | 5 (6) | 12 (8) | 5 (7) |
| $50,000 to $74,999 | 41 (12) | 10 (11) | 19 (12) | 8 (11) |
| $75,000 to $99,999 | 60 (17) | 16 (18) | 28 (18) | 11 (15) |
| $100,000–149,999 (NET) | 148 (43) | 43 (49) | 60 (38) | 37 (49) |
| $100,000 to $124,999 | 83 (24) | 22 (25) | 35 (22) | 19 (25) |
| $125,000 to $149,999 | 65 (19) | 21 (24) | 25 (16) | 18 (24) |
| $150,000 to $199,999 (NET) | 26 (8) | 6 (7) | 10 (6) | 7 (9) |
| $200,000 or more (NET) | 21 (6) | 0 | 16 (10) | 1 (1) |
| $200,000 to $249,999 | 8 (2) | 0 | 5 (3) | 1 (1) |
| $250,000 or more | 13 (4) | 0 | 11 (7) | 0 |
| **Marital status, *n* (%)** | |  |  |  |
| Married/living with partner | 276 (80) | 69 (79) | 125 (79) | 62 (83) |
| Divorced/separated/widowed | 51 (15) | 13 (15) | 24 (15) | 11 (15) |
| **Highest education level, *n* (%)** | | |  |  |
| Less than high  school diploma | 25 (7) | 7 (8) | 4 (3) | 7 (9) |
| High school diploma | 158 (46) | 40 (46) | 58 (37) | 54 (72) |
| 4-year college degree  or more | 161 (47) | 40 (46) | 96 (61) | 14 (19) |
| **Employment status, *n* (%)** |  |  |  |  |
| Employed | 121 (35) | 23 (26) | 59 (37) | 29 (39) |
| Not employed | 223 (65) | 64 (74) | 99 (63) | 46 (61) |
| **Parental status, *n* (%)** | | | | |
| Parent | 272 (79) | 69 (79) | 121 (77) | 68 (91) |
| Not a parent/legal guardian | 71 (21) | 17 (20) | 37 (23) | 7 (9) |
| **Household size, *n* (%)** | | | |  |
| 1 member | 12 (3) | 2 (2) | 8 (5) | 1 (1) |
| 2 members | 57 (17) | 5 (6) | 44 (28) | 5 (7) |
| 3 members | 65 (19) | 21 (24) | 28 (18) | 9 (12) |
| 4 members | 93 (27) | 29 (33) | 36 (23) | 22 (29) |
| 5+ members | 117 (34) | 30 (34) | 42 (27) | 38 (51) |
| **Time taken to get to treatment center, *n* (%)** | | | |  |
| 0–9 minutes | 3 (1) | 0 | 3 (2) | 0 |
| 10–19 minutes | 39 (11) | 5 (6) | 21 (13) | 12 (16) |
| 20–29 minutes | 32 (9) | 3 (3) | 17 (11) | 11 (15) |
| 30–39 minutes | 78 (23) | 20 (23) | 36 (23) | 16 (21) |
| 40–49 minutes | 73 (21) | 22 (25) | 23 (15) | 21 (28) |
| 50–59 minutes | 26 (8) | 8 (9) | 12 (8) | 4 (5) |
| ≥60 minutes | 93 (27) | 29 (33) | 46 (29) | 11 (15) |
| **Method of transport used to get to appointments, *n* (%)** | | | | |
| Friend or family  member drives | 196 (57) | 42 (48) | 89 (56) | 51 (68) |
| Ride-share service or taxi | 104 (30) | 35 (40) | 40 (25) | 19 (25) |
| Respondent drives | 79 (23) | 11 (13) | 57 (36) | 9 (12) |
| Public transportation | 75 (22) | 31 (36) | 28 (18) | 11 (15) |
| Other | 2 (1) | 0 | 2 (1) | 0 |
| **Medical practice providing treatment, *n* (%)** | | | | |
| National cancer center | 129 (38) | 37 (43) | 55 (35) | 23 (31) |
| Community cancer center | 82 (24) | 26 (30) | 35 (22) | 15 (20) |
| Community hospital | 65 (19) | 19 (22) | 25 (16) | 20 (27) |
| University hospital | 34 (10) | 3 (3) | 22 (14) | 6 (8) |
| Private practice | 27 (8) | 2 (2) | 15 (9) | 10 (13) |
| Unsure | 2 (1) | 0 | 1 (1) | 1 (1) |
| Currently not  receiving treatment | 5 (1) | 0 | 5 (3) | 0 |

*Base sizes are <100; therefore, results should only be interpreted directionally. ^†^Among Hispanic respondents, 16% (*n* = 12) also identified as White and 72% (*n* = 54) as Black/African American.

**Supplementary Figure 1. Flowchart of survey respondents.**

*PAGs included: BLKHLTH, COLONTOWN, Colorectal Cancer Alliance, and Family Reach.

PAGs, patient advocacy groups.

**Supplementary Figure 2.** **Respondent attitudes towards diagnosis, supportive resources, experiences, and treatment decision-making during their mCRC journey*.**

*Percentages may not add to 100% due to rounding.
mCRC, metastatic colorectal cancer.

**Supplementary Figure 3. Factors that respondents felt had a negative impact on their ability to receive necessary mCRC care*.**

*Respondents could select more than one response.
mCRC, metastatic colorectal cancer.

**Supplementary Figure 4. Factors that respondents reported would make them feel heard, seen, and valued when interacting with their mCRC healthcare team*.**

*Respondents could select more than one response; excludes ‘not applicable’ responses.
mCRC, metastatic colorectal cancer.
